# Linking Myocardial Infarction and Frailty Status at Old Age in Europe: Moderation Effects of Country and Gender

**DOI:** 10.3390/jcdd11060176

**Published:** 2024-06-08

**Authors:** Trinidad Sentandreu-Mañó, Zaira Torres, Cecilia Luján-Arribas, José M. Tomás, José Javier González-Cervantes, Elena Marques-Sule

**Affiliations:** 1 Department of Physiotherapy, University of Valencia, 46010 Valencia, Spain; trinidad.sentandreu@uv.es (T.S.-M.); celua@alumni.uv.es (C.L.-A.); elena.marques@uv.es (E.M.-S.); 2 Department of Methodology for the Behavioral Sciences, University of Valencia, 46010 Valencia, Spain; zaira.torres@uv.es; 3 Department of Nursing, European University, 46010 Valencia, Spain; josejavier.gonzalez@universidadeuropea.es

**Keywords:** ischemic heart disease, myocardial infarction, frailty, gender, European older adults

## Abstract

Background: Myocardial infarction (MI) is a serious condition affecting a considerable number of individuals, with important clinical consequences. Understanding the associated factors is crucial for effective management and prevention. This study aimed to (1) examine the association between MI and frailty in a sample of older European adults and (2) investigate the moderating effects of country and gender on this association. Methods: A cross-sectional survey of 22,356 Europeans aged 60 years and older was conducted. The data come from the sixth wave of the Survey of Health, Ageing and Retirement in Europe. Frailty, MI, gender, and country were studied. Results: Frailty is strongly associated with MI. Robust older adults are 13.31 times more likely not to have an MI. However, these odds drop to 5.09 if pre-frail and to 2.73 if frail. Gender, but not country, moderates this relationship. There is a strong association between MI and frailty in men, whereas for women, the association is not as strong. Conclusions: Frailty is highly associated with MI in European older adults. Country did not moderate the link between frailty and MI but gender does, with the relationship being notably stronger in men. The frailty–MI association remained significant even when controlling for a number of personal conditions and comorbidities.

## 1. Introduction

Cardiovascular diseases (CVDs) cause approximately one-third of deaths worldwide [1]. Among CVDs, ischemic heart disease (IHD) is the most prevalent [2]. IHD is a heart condition resulting from pathophysiological changes secondary to an imbalance between oxygen demand and the supply of cardiac muscle [3]. The most severe clinical manifestation of IHD is myocardial infarction [4]. Myocardial infarction (MI) is the result of a decrease in or complete cessation of blood flow to a portion of the myocardium that can be a silent occurrence that goes unnoticed or may manifest as a catastrophic event, leading to hemodynamic deterioration and sudden death [5]. It is estimated that more than three million individuals are affected by MI each year [6]. While mortality from MI has significantly decreased in recent years [7,8], the incidence of MI in developed countries remains remarkably high. Moreover, individuals now live longer with chronic CVD [9,10]. The incidence of IHD is expected to continue rising, driven not only by the increasing rates of obesity, diabetes, and metabolic syndrome but also by the aging population [11]. Age is a cardiovascular risk factor that may be increased by additional factors, among which frailty plays a significant role [12].

Physical frailty is defined as a complex clinical state characterized by various signs and symptoms, including unintentional weight loss, self-reported exhaustion, slow walking speed, low energy expenditure, and weak grip strength [13]. Frailty is characterized by a decline in the physiological capacity of multiple organ systems and is associated with disability, institutionalization (retirement homes), and mortality [14,15]. The coexistence of CVD and frailty is common in aging populations [12,16]. Frailty exacerbates and intensifies the cardiovascular risk factors linked to the onset of older age [17]. The presence of frailty syndrome not only accelerates the onset of any type of CVD but also multiplies the risk of death from cardiovascular causes by approximately fourfold [18]. Various conditions, including low physical activity, poor nutrition, diabetes, and hypertension, can increase the risk of both CVD and frailty. These conditions often interact, further increasing the risk of adverse outcomes [12]. The precise pathways linking frailty and CVD are unknown, but one possible explanation may be “inflammaging,” in which aging tissues seem to elevate proinflammatory markers. High levels of these markers in the blood are associated with muscle weakness, reduced muscle mass, poor function, and limited mobility, among others, which increase the risk of frailty and CVD [19]. 

In the context of acute MI, prior studies have underscored the significance of frailty as a crucial prognostic indicator for adverse outcomes such as rehospitalization, bleeding, and mortality, among others [20,21,22]. Nevertheless, there is limited evidence regarding the association between frailty and MI. Additionally, disparities in cardiac problems and frailty exist among European countries. Mortality resulting from CVDs is notably higher in middle-income countries compared to high-income countries [10,23]. The prevalence of IHD is more pronounced in Eastern European countries compared to their Western counterparts [10]. Additionally, Southern and Eastern European countries exhibit higher rates of frailty, while Western and Northern Europe report the lowest frailty rates [24].

Finally, another key factor to consider when examining the relationship between MI and frailty is gender. The incidence of MI is significantly higher in men than in women [25]. However, once acute MI is established, mortality rates tend to be higher in women [26,27]. In addition, a robust relationship between gender and frailty has been found, with several studies indicating that European women tend to exhibit higher levels of frailty compared to men [28]. Despite women having a longer life expectancy, they are observed to be more frail than men [28,29], a phenomenon known as the gender–frailty paradox. 

National and international organizations have recognized CVDs among frail individuals as a crucial area for healthcare attention, requiring careful clinical decision-making [30]. However, there is a lack of comprehensive recommendations in this regard [31]. Exploring the relationship between frailty and MI, as well as the underlying conditions that link them, may be relevant for both treatment and prevention. For this reason, the aims of this study were (1) to associate MI and frailty in a sample of European older adults; (2) to investigate the moderating effects of country and gender on this association; and, additionally, (3) to further analyze the association between frailty and MI in a multivariate context while controlling for several personal variables and comorbidities.

## 2. Materials and Methods

### 2.1. Study Design and Participants

A cross-sectional study was carried out using the 6th wave of the Survey of Health, Ageing and Retirement in Europe (SHARE) [32,33]. This project has a probability-based panel survey design [34] targeting respondents aged 50 and older across 26 European countries and Israel. The SHARE project has been approved by the Ethics Committee of the Max Planck Society for the Advancement of Science (Approval Code: 2021_24). SHARE country implementations were reviewed and approved by the respective ethics committees or institutional review boards. Participation in SHARE is voluntary, confidential, and based on informed consent. The collection and use of SHARE data adhere to the European General Data Protection Regulation. The data are exclusively for scientific use and are safeguarded by the German Federal Statistics Act and the German Federal Data Protection Law. For the purposes of the present study, the sample involved 22,356 participants aged 60 years and older belonging to six European countries: Sweden, Spain, France, Greece, Czech Republic, and Estonia.

### 2.2. Variables

Frailty was measured using SHARE’s operationalized version [35,36] which is based on the five physical criteria established by Fried [13]. The SHARE operationalized frailty phenotype has been validated by different authors [37,38,39]. The specific measurements included the following:

- Fatigue was measured using the following question: “In the last month, have you had little energy to do the things you wanted to do?” with a dichotomous answer of “yes” or “no”.
- Weakness was recorded from the highest of four consecutive dynamometer measurements of grip strength, making two in each hand, and applying the limits of gender and body mass index cutoffs set by Fried et al.
- Unintentional weight loss was recorded by reporting “a diminution in desire for food” in response to the question “How has your appetite been like?” or, in the case of an uncodable response to this question, by answering “less” to the question “So, have you been eating more or less than usual”?
- Physical activity was assessed in participants responding: “How often do you engage in activities that require a low or moderate level of energy, such as gardening, cleaning the car or going for a walk?” This criterion was fulfilled when the answer was “one to three times a month”, “hardly ever”, or “never”.
- Slowness was recorded as a positive answer to any of the following questions: “Because of a health problem, do you have difficulty walking 100 m?” and “Because of a health problem, do you have difficulty climbing one flight of stairs without resting?” The difficulties are expected to last more than 3 months.

One point was allocated for each fulfilled criterion, following the guidelines of Fried [13]. Participants with zero points were classified as “robust”, those with one or two points as “pre-frail”, and those with three to five points were classified as “frail”.

Myocardial infarction was measured with the question “Has a doctor ever told you that you had a heart attack?” Response options were 1 “yes” or 0 “no”. 

We also measured some personal conditions: gender, age, and body mass index (BMI). Gender was a dichotomous variable registered as female = 1 and male = 0. Age was measured in years. BMI was calculated as a person’s weight in kilograms divided by the square of height in meters.

Finally, several comorbidities were also included as control variables: number of chronic diseases; whether the person has suffered (1) or not suffered (0) a stroke; whether the person stayed overnight in hospital last 12 months (1) or not; physical inactivity measured as whether the person does neither vigorous nor moderate physical activity (1) or yes (0); whether the person takes drugs for high blood cholesterol (1) or not (0); whether the person takes drugs for high blood pressure (1) or not (0); and whether the person takes drugs for coronary diseases (1) or not (0). 

The six European countries were selected based on geographical location, as well as a typology of European welfare regimes [40,41]: Sweden from Northern Europe; Czech Republic and Estonia from Eastern Europe/post-communist countries; Greece and Spain from Southern Europe, and France from Western Europe. 

### 2.3. Statistical Analyses

Statistical analyses included descriptive statistics for all the variables under study. In order to test the bivariate association between MI and frailty status, a chi-square test of independence together with Cramer’s V as a measure of effect size was estimated. Additionally, the moderation effects of gender and country on the association between MI and frailty were analyzed with log-linear models. Log-linear models are a Generalized Linear Model appropriate when the goal of research is to determine if there is a statistically significant relationship among three or more discrete variables. It represents expected cell counts as functions of row and column effects and interactions, making no distinction between response and explanatory variables [42]. Finally, a binary logistic regression was estimated to predict MI. The predictors were personal characteristics (age, gender, BMI, and physical inactivity), clinical conditions and medical comorbidities (number of chronic diseases, staying in hospital, having a stroke, and taking drugs for blood cholesterol, blood pressure, or coronary diseases), and frailty. The aim of this logistic regression was to analyze the predictive impact frailty has on MI while the other predictors are controlled for. All these statistical analyses were estimated in R [43]. Specifically, the packages employed were gmodels [44], vcd [45], MAAS, and DescTools [46].

## 3. Results

### 3.1. Descriptive Statistics

The sample included 22,356 older adults. Among these, 43.7% were male and 56.3% were female. The mean age was 71.96 (SD = 8.27) years, with a minimum age of 60 and a maximum of 105 years. The average years of education were 10.48 (SD = 4.32). Of the total of sample, 14.8% suffered an MI, while 7744 (34.6%) were classified as robust, 10,985 (49.1%) as pre-frail, and 3627 (16.2%) as frail. The characteristics of the sample are shown in Table 1.

Table 2 summarizes the incidence of myocardial infarction by country. Estonia has the highest percentage of people diagnosed with MI (20.9%), while Sweden has the lowest percentage (11.6%).

### 3.2. Relationship between Frailty and Myocardial Infarction

The association between frailty status and MI in the overall sample of Europeans was analyzed with a chi-square test of independence. This chi-square was statistically significant (χ^2^ (2) = 805.26, *p* < 0.0001, Cramer’s V = 0.189, 95% CI [0.176, 0.203]), and the effect size may be considered small/medium. The odds of not having an MI against having an MI in robust older adults are 13.31. That is, robust adults are 13.31 times more likely not to suffer an MI. However, those odds drop to 5.09 in pre-frail older adults and drop even more to 2.73 in frail ones. Therefore, frailty is a significant risk factor for MI.

Table 3 shows information about the cross-tabulation of MI and frailty status. It provides information about the counts in each cell, the row percent, and the standardized residuals. 

**Figure 1:**
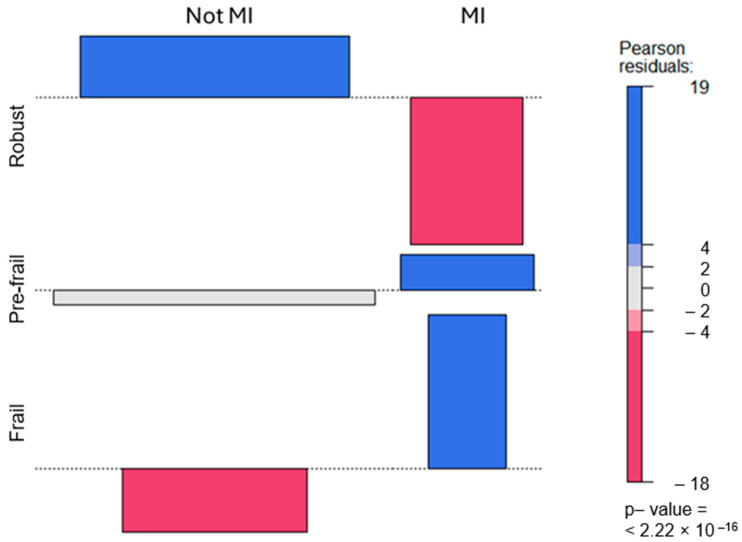
Standardized residuals for the cross-classification of myocardial infarction (MI) and frailty. Note: blue cells indicate more likely than expected, and red cells indicate less likelihood than expected; MI = myocardial infarction; *p*-value < 0.05 is statistically significant.

Standardized residuals superior to the absolute value of 2 may be considered statistically significant. A good way to assess the degree of association between the two variables (MI and frailty) is through a graphical representation of the standardized residuals. This graph is presented in Figure 1. This figure shows in a blue color those cells that are more likely than expected, whereas the red color indicates less likelihood than expected. Therefore, it is clear that robust older adults are much less likely to suffer an MI than pre-frail and frail older adults, and it is also shown that the likelihood of suffering an MI increases from pre-frail to frail status.

### 3.3. Moderation by Gender and Country

A secondary aim of this research was to test if the overall association found between MI and frailty was moderated (conditioned) by country and gender. In order to test for these two moderations effects, we have estimated two log-linear models. The log-linear models were estimated backwards. 

The first set of log-linear models aimed at analyzing the moderation effect of country. We started with the saturated model with all main effects, all two-way interactions, and the three-way interaction. These models are nested and therefore may be compared with Likelihood Ratio Tests comparing two models. Fit indexes for all models are presented in Table 4. When country moderates the MI–frailty association, the fit indexes show that two-way associations are needed, but the three-way interaction is not relevant, as the Akaike Information Criterion (AIC) and Bayesian Information Criterion (BIC) for this model increased indeed. In other words, country does not moderate the association between MI and frailty. This conclusion is further supported by the comparison of the Likelihood Ratio Tests. The saturated model (three-way interactions) compared with the two-way interactions was not statistically significant (χ^2^(10) = 12.4, *p* = 0.260), and therefore, a three-way interaction does not add model fit. However, the two-way interactions compared to the main effects (only) model was statistically significant (χ^2^(17) = 1570.2, *p* < 0.001), indicating that these interactions are statistically relevant.

**Figure 2:**
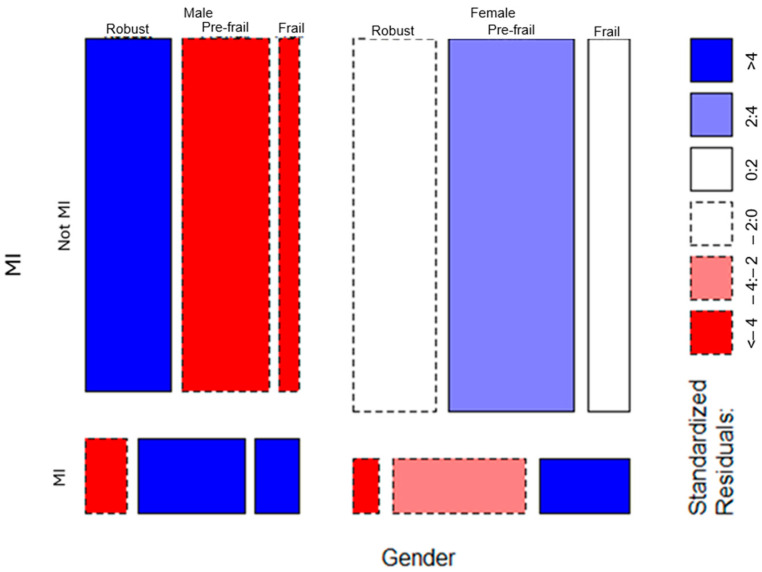
Mosaic plot showing the standardized residuals for the three-way interaction of myocardial infarction (MI), frailty, and gender. Note: blue cells indicate more likely than expected, and red cells indicate less likelihood than expected; MI = myocardial infarction.

Regarding the moderating effect of gender, the set of log-linear models had fit indexes (see Table 4) that pointed out that the association of MI and frailty is conditional on gender. The AIC and BIC values went down when the three-way interaction was considered. Further evidence about the moderating effect of gender comes from the comparison of the saturated model (three-way interaction) and the model with only the two-way interactions. This comparison is statistically significant (χ^2^(2) = 13, *p* < 0.001), as it is the comparison between the model with two-way interactions and the main effects model (χ^2^(5) = 1570.2, *p* < 0.001). All in all, indexes and tests show that gender conditions the association of MI and frailty. Calculating standardized residuals and presenting them in a graphical way may help to understand the nature of this moderation effect. The mosaic plot in Figure 2 shows standardized residuals. Again, the blue color represents more likelihood than expected in a particular cell, and, on the contrary, the red color represents a lower probability than expected for that cell. We can see that for men there is an increased likelihood of suffering an MI as they became pre-frail and frail. That is, there is a clear and strong association between MI and frailty for men. However, this association, although present in women, is not so strong, as the amount of the standardized residuals demonstrates. In sum, the link between frailty and MI is much stronger for European men than for European women. 

### 3.4. Multivariate Prediction of Myocardial Infarction

A binary logistic regression was estimated to predict MI. The predictors were personal characteristics (age, gender, BMI, and physical inactivity), clinical conditions and medical comorbidities (number of chronic diseases, staying in hospital, having a stroke, and taking drugs for blood cholesterol, blood pressure, or coronary diseases), and frailty. The aim of this logistic regression was to analyze the predictive impact frailty has on MI while the other predictors are controlled for. Table 5 offers the model’s estimates and *p* levels as well as OR for all predictors.

All predictors are able to predict 37.5% of the variance in MI (Nagelkerke’s R-square = 0.375). All the control variables (personal characteristics, clinical conditions, and comorbidities) were significantly related to MI with the exception of BMI. Regarding the effects of frailty status on MI, they remained statistically significant even after controlling for the rest of the predictors. As can be seen in Table 5, on one hand, being pre-fragile increases the probability of suffering an MI by 39%, and on the other hand, being frail increases the probability of suffering an MI by 56%.

## 4. Discussion

To the best of our knowledge, this is the first study linking MI and frailty in a European sample and assessing the moderating effects of country and gender. The study findings underscore the importance of considering frailty as a potential associated factor for MI among the European population. The robustness of individuals appears to have a protective effect against MI, as evidenced by the substantially higher odds of not experiencing an MI in robust individuals compared to those who are pre-frail or frail. Conversely, frail individuals have a greater probability of suffering an MI. These results are in line with the previous literature, indicating a higher risk of more rapid onset of any type of CVD in frail and pre-frail people, even after accounting for age, gender, and other cardiovascular risk factors [16].

Particularly for MI, in a study involving Australian patients, it was found that around one in six older patients with ST-segment elevation MI and one in three older patients with non-ST-segment elevation MI were considered frail. Even after adjusting for traditional risk factors, increased frailty was associated with higher rates of all-cause mortality during hospitalization and in the midterm post-discharge period [47]. A recent meta-analysis showed that the prevalence of frailty increased among older patients with acute MI undergoing percutaneous coronary intervention (PCI), particularly in those with ST-segment elevation MI. In addition, frail older adults with acute MI undergoing PCI were more likely to experience worse clinical outcomes, such as death, bleeding, and rehospitalization [21]. Finally, another meta-analysis reported a strong association between frailty and bleeding and mortality in older adults with MI, recommending frailty assessment to be considered as an additional risk factor [48]. Our findings have notable implications for clinical practice. Healthcare providers should recognize frailty as a potential marker for increased MI risk and incorporate frailty assessments into routine clinical evaluations, particularly among older adults. Early identification of frail individuals could facilitate targeted interventions aimed at reducing cardiovascular events and improving overall health outcomes. However, the potential benefit that this could yield remains uncertain and requires future research [31].

Despite the previous literature highlighting disparities in the prevalence of cardiac problems [10] and frailty rates [24] among European countries, our log-linear model analysis revealed that country does not act as a moderator in the association between MI and frailty. There is limited literature investigating the moderating effects of country on the relationship between MI and frailty. Thus, our study, by concluding that there is no moderation, is pioneering in shedding light on this underexplored aspect.

Gender is another major risk factor regarding the onset, manifestation, and management of CVDs in older adults [49]. These differences that lead to discrepancies in CVD risk factors and outcomes between men and women could be largely attributed to sex hormones and their associated receptors [50]. According to the American Heart Association, IHD is more common in older men than in older women. When it comes to MI, rates of diagnosis are higher in older men compared to older women [51]. In addition, a large body of literature shows that frailty status is more common in women, but in contrast, higher mortality among men results from frailty [36,52]. Our findings show an effect of gender in moderating the relationship between MI and frailty, revealing that European men exhibit a stronger association between MI and frailty compared to European women. It has been reported that women are more likely to have pre-existing diabetes, hypertension, and stroke prior to presenting with MI in comparison to men. Conversely, women experience lower odds of dyslipidemia, angina and IHD, and smoking history. Even for shared risk factors, the prevalence and associated risk tend to vary [53]. These observed relationships likely reflect a complex interplay among biological, social, and cultural factors [54,55]. This study emphasizes the importance of considering gender-specific approaches in addressing frailty and MI prevention and management. 

Additionally, our findings demonstrate that cardiovascular risk factors such as physical inactivity, high blood pressure, and cholesterol are significantly associated with MI, in line with the extensive evidence available on the subject [56,57]. Controversially, BMI does not appear to be a potential predictor of MI in the analyzed model. This lack of association may be age-dependent, differing mainly between profiles of younger individuals compared to older ones [58,59], or simply its effect disappears when other cardiovascular risk factors are controlled for. Moreover, it may be more closely related to the outcome measure used, where the waist-to-hip ratio, not BMI, demonstrated a significant association with MI risk [60]. Therefore, further investigation into the relationship between adiposity and MI is necessary [61]. However, other cardiovascular diseases and associated comorbidity are also linked with MI, as expected, since they may share underlying risk factors and pathophysiological mechanisms [62,63].

The strengths of this study are the use of a large sample and representative data from several European countries. Nonetheless, several limitations should be acknowledged. One of the main limitations is the cross-sectional nature of the study, which does not allow studying the direction of causality. Additionally, the reliance on self-reported scales introduces the possibility of self-report bias and issues related to common method variance, potentially compromising the accuracy of the data. Furthermore, although we focused on the moderating effects of gender and country, it is important to acknowledge that other variables, such as previous hospitalizations or drug use [16], may also significantly influence the outcomes. These limitations should be carefully considered when interpreting the findings. 

Further research should explore the underlying mechanisms linking frailty to increased MI risk and identify potential intervention strategies to mitigate this risk with a gender perspective. Longitudinal studies could provide valuable insights into the temporal relationship between frailty and MI, while clinical trials could assess the effectiveness of targeted interventions in reducing cardiac events among frail individuals.

## 5. Conclusions

Frailty is a factor associated with MI among older adults in Europe. While country does not play a moderating role, gender conditions this association, with the relationship being notably stronger in European men. This study underscores the importance of addressing frailty as a preventive measure against MI and emphasizes the need for gender-specific approaches in both research and clinical interventions aimed at reducing cardiac events.

## Figures and Tables

**Table 1:** Descriptive statistics (*n* = 22,356).

Variable	Mean ± SD o *n* (%)
Age	71.96 ± 8.27
Gender	
Male	9766 (43.7)
Female	12,590 (56.3)
Country	
Sweden	3397 (15.2)
Spain	4497 (20.1)
France	2861 (12.8)
Greece	3558 (15.9)
Czech Republic	3896 (17.4)
Estonia	4147 (18.5)
Marital status	
Married, living together with spouse	14,316 (64.7)
Registered partnership	231 (1.0)
Married, living separated from spouse	240 (1.1)
Never married	1036 (4.7)
Divorced	1735 (7.8)
Widowed	4581 (20.7)
Years of education	10.48 ± 4.32
Body mass index	27.31 ± 4.57
Number of comorbid conditions	2.06 ± 1.65
Myocardial infarction	3307 (14.8)
High blood pressure or hypertension	10705 (47.9)
High blood cholesterol	5912 (26.4)
Diabetes or high blood sugar	3777 (16.9)
Frailty	
Robust	7744 (34.6)
Pre-frail	10,985 (49.1)
Frail	3627 (16.2)

SD = Standard deviation.

**Table 2:** Frequency of myocardial infarction by country.

Country	*n* (%)
Sweden	394 (11.6%)
Spain	543 (12.2%)
France	446 (15.6%)
Greece	506 (14.3%)
Czech Republic	555 (14.3%)
Estonia	865 (20.9%)

**Table 3:** Cross-table of occurrence of myocardial infarction (MI) and frailty status.

		Not MI	MI
	Count	7166	538
Robust	Row percent	93.017%	6.983%
	Standardized residual	7.461	−17.881
	Count	9174	1799
Pre-frail	Row percent	83.605%	16.395%
	Standardized residual	−1.778	4.261
	Count	2655	970
Frail	Row percent	73.241%	26.759%
	Standardized residual	−7.783	18.654

**Table 4:** Fit indexes for the set of log-linear models.

	Effects Included	Deviance	AIC	BIC
	Main	1582.6	1880	1894
Country moderates	Main + two-way interactions	12.4	344	385
	Main + two-way + three-way interactions	0	352	409
	Main	1369.4	1486	1488
Gender moderates	Main + two-way interactions	13	139	144
	Main + two-way + three-way interactions	0	130	136

Note: AIC = Akaike Information Criterion; BIC = Bayesian Information Criterion.

**Table 5:** Binary logistic regression to predict myocardial infarction with personal characteristics, comorbidities, and frailty.

			95% CI	
Predictor	*p*	OR	Lower	Upper
Constant	<0.001	0.01	0.00	0.01
Age	<0.001	1.02	1.01	1.03
Female vs. male	<0.001	0.51	0.46	0.56
Body mass index	0.927	0.99	0.98	1.01
Physically inactive vs. active	<0.001	0.74	0.65	0.85
Number of chronic diseases	<0.001	1.79	1.73	1.85
Staying in hospital vs. not	<0.001	0.65	0.59	0.73
Stroke vs. not	<0.001	0.28	0.23	0.34
Blood cholesterol vs. not	0.034	0.89	0.81	0.99
Blood pressure vs. not	0.002	0.84	0.76	0.93
Coronary disease vs. not	<0.001	9.37	8.38	10.47
Frailty				
Pre-frail vs. robust	<0.001	1.39	1.23	1.57
Frail vs. robust	<0.001	1.56	1.34	1.83

Note: OR = Odds Ratio; CI = Confident Interval; *p*-value < 0.05 is statistically significant.

## Data Availability

The data that support the findings of this study are available at the SHARE Research Data Center to the entire research community free of charge (www.share-project.org). Restrictions apply to the availability of these data, which were used under license for the current study and, thus, are not publicly available. Data are, however, available from the authors upon reasonable request and with permission of the SHARE Project (https://share-eric.eu/data/data-access).
